# The Structure and Function of Paraoxonase-1 and Its Comparison to Paraoxonase-2 and -3

**DOI:** 10.3390/molecules25245980

**Published:** 2020-12-17

**Authors:** Ajda Taler-Verčič, Marko Goličnik, Aljoša Bavec

**Affiliations:** Institute of Biochemistry and Molecular Genetics, Faculty of Medicine, University of Ljubljana, Vrazov trg 2, 1000 Ljubljana, Slovenia; ajda.taler-vercic@mf.uni-lj.si (A.T.-V.); marko.golicnik@mf.uni-lj.si (M.G.)

**Keywords:** PON1, PON2, PON3, paraoxonase, lactonase, arylesterase, organophosphate, structure, kinetic, oxidative stress, atherosclerosis

## Abstract

Serum paraoxonase-1 (PON1) is the most studied member of the group of paraoxonases (PONs). This enzyme possesses three enzymatic activities: lactonase, arylesterase, and paraoxonase activity. PON1 and its isoforms play an important role in drug metabolism as well as in the prevention of cardiovascular and neurodegenerative diseases. Although all three members of the PON family have the same origin and very similar amino acid sequences, they have different functions and are found in different locations. PONs exhibit substrate promiscuity, and their true physiological substrates are still not known. However, possible substrates include homocysteine thiolactone, an analogue of natural quorum-sensing molecules, and the recently discovered derivatives of arachidonic acid—bioactive δ-lactones. Directed evolution, site-directed mutagenesis, and kinetic studies provide comprehensive insights into the active site and catalytic mechanism of PON1. However, there is still a whole world of mystery waiting to be discovered, which would elucidate the substrate promiscuity of a group of enzymes that are so similar in their evolution and sequence yet so distinct in their function.

## 1. Introduction

Paraoxonases are a group of three identified enzymes: paraoxonase-1 (PON1, EC 3.1.1.2, 3.1.1.81, 3.1.8.1), paraoxonase-2 (PON2, EC 3.1.1.2, 3.1.1.81), and paraoxonase-3 (PON3, EC 3.1.1.2, 3.1.1.81, 3.1.8.1). The most studied among them is PON1. However, it is not necessarily considered the most important. The oldest member appears to be PON2, from which first PON3 and then PON1 evolved. Although PONs are very similar in their amino acid sequences, they have different functions and are found at different locations. PON1 and PON3 reside in high-density lipoproteins (HDLs) in the blood system and exclusively hydrolyze organophosphates and bulky statin lactones, respectively, whereas PON2 is found in many tissues and mainly acts as an intracellular protector against oxidative stress. PON1 isoforms play an important role in drug metabolism and the prevention of cardiovascular and neurodegenerative diseases. All three PONs exhibit a broad range of enzymatic activities towards various types of substrates in a single active site. PON2 exhibits lactonase and very low arylesterase activity. PON3 exhibits high lactonase, weak arylesterase, and almost no paraoxonase activity. PON1 exhibits all three activities.

## 2. Paraoxonase 1: An Evolutionary Highlight with Many Enzymatic Activities

PON1 was the first studied member of the PON family. Initially, it was referred to as A esterase, but was later named PON because of its almost exclusive ability to hydrolyze paraoxon, the oxon form of the insecticide parathion [1,2,3]. PON1 is a 43 kDa calcium-dependent glycoprotein with 355 amino acid residues [4,5]. After being synthesized in the liver, PON1 is released into the circulation and found mainly in HDLs and, to a lesser extent, in very low-density lipoproteins and chylomicrons [6]. Moreover, PON1, myeloperoxidase, and HDLs form a functional complex by binding to each other [7]. Free PON1 has lower enzymatic activity than HDL-bound PON1. PON1 is transported from the liver to several tissues [8] in which it binds to cell membranes and protects lipids against peroxidation [6]. Furthermore, PON1 prevents low-density lipoprotein (LDL) oxidation and impairs the inflammatory response [9].

The second important role of PON1 is protecting against specific organophosphate (OP) exposure. Studies on knockout mice (PON1^−/−^) have shown that PON1 is an important protector against exposure to the OPs chlorpyrifos oxon and diazoxon, but not paraoxon [10]. In addition, newborn humans express only one third of the PON1 levels that adults express and need up to two years to reach adult concentrations of PON1, indicating an increased sensitivity of toddlers to OP exposure [11]. Therefore, constructing more catalytically efficient variants of PON1 will be required for treating OP poisoning.

Another protective role of PON1 is its hydrolysis of homocysteine thiolactone (HCTL). HCTL is a toxic metabolite that interacts with lysines and modifies proteins, which leads to protein inactivation and dysfunction. Elevated blood concentrations of HCTL are associated with an increased risk of developing cardiovascular, neurological and autoimmune diseases as well as cancer [12,13,14,15]. However, the physiological relevance of PON1 regarding HCTL is questionable due to its very low specific enzyme activity [16].

The enzymatic activities of PON1 include lactonase, thiolactonase, arylesterase and aryldialkylphosphatase activities. The latter are commonly known as paraoxonase, phosphotriesterase or organophosphatase activities. PON1 activity-related functions include the above-mentioned clearance of OPs (e.g., insecticides and nerve agents) [17], involvement in drug metabolism (both drug activation and inactivation) [18] and a protective role in atherosclerosis by reacting with proinflammatory oxidized lipids that are present in LDLs [10].

Structure-reactivity studies on PON1 with an impressive list of OPs, aryl esters and lactones suggest that its native activity is lactonase [19]. PON1 is a less potent lactonase than PON3 but it hydrolyzes a much broader spectrum of lactones at higher rates than PON3 [4]. Furthermore, in human serum, PON1 activity towards different lactones and other substrates varies according to its polymorphic forms (isoforms). PON1 has two important polymorphic sites at amino acid residue positions 55 and 192. The substitution of leucine at position 55 by methionine (L55M) increases paraoxonase activity [20] and results in enzyme concentration differences between M and L isoforms [21]. The substitution of glutamine at position 192 with arginine (Q192R) leads to an increase in lactonase and paraoxonase activity and substrate affinity for dihydrocoumarin (DHC) and paraoxon, respectively, and decreases arylesterase activity and increases substrate affinity for phenyl acetate (PA) [16]. Moreover, the thiolactonase activity of PON1 is also affected by substitutions at positions 55 and 192. PON1, carrying both L55 and R192, hydrolyzes HCTL more efficiently than the isoform with M55 and Q192 [22]. PON1 isoforms with interindividual variations in enzyme activity have a great impact on individual health and the development of different diseases and medical conditions.

Research started to focus on PON1 after terrorists released sarin in a Tokyo subway in 1995, which resulted in several deaths [17]. Scientists searched for a potent enzyme for the rapid clearance of nerve agents [23]. Among butyrylcholinesterase, acetylcholinesterase, carboxylesterase and PON1, the latter exhibited the most prominent enzyme activity, which could be further improved by introducing point mutations into its active site [23,24]. So far, the naturally present enzyme in human plasma enables protection from long-term small-dose exposure to OPs (insecticides). However, the amounts needed to neutralize nerve agents are still too high to enable its commercial use [23].

## 3. The Catalytic Versatility of PON1

**Figure 1:**
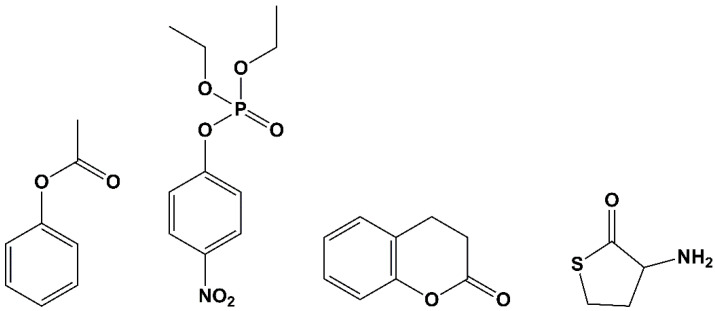
PON1 substrates from left to right: phenyl acetate, paraoxon, dihydrocoumarin and homocysteine thiolactone.

Although human PON1 has been shown to exhibit multiple hydrolytic activities, the physiological substrate(s) for the enzyme is not known. The enzyme is capable of hydrolyzing various types of substrate molecules, e.g., aryl esters, phosphotriesters, lactones and thiolactones [16,25]. Thus, its activities can be broadly grouped into three categories: arylesterase, paraoxonase, and (thio)lactonase activities [19]. However, several reports suggest that the catalytic activity of native PON1 is lactonase activity [19,26,27]. Here, we show some of the most studied substrates of PON1 (Figure 1).

**Figure 2:**
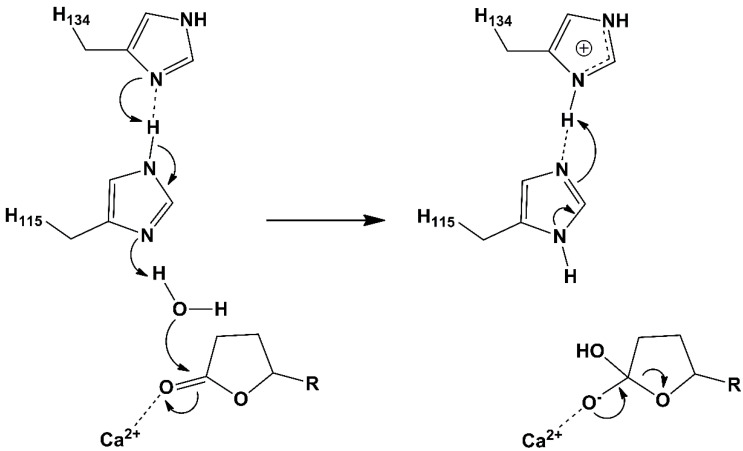
Presumed proton transfer through a histidine (H115/H134) dyad for water molecule activation, which is followed by tetrahedral intermediate (adduct) formation at the carbonyl groups of aryl esters or (thio)lactones. Although the negative charge of the elusive high-energy tetrahedral intermediate is electrostatically stabilized by the catalytic calcium ion, the C-O bond of this short-life species is subsequently cleaved, and the hydrolytic product is formed.

PON1 has appreciable arylesterase activity, with PA being a typical substrate with a catalytic efficiency of k_cat_/K_M_ ≈ 10^6^ M^−1^ s^−1^ [19]. The crystal structure of recombinant PON1 (rePON1) also provides insight into the catalytic center of the enzyme [28]. One of two calcium ions lies at the bottom of the active site and is postulated to play an electrostatic role in catalysis by stabilizing negative charges of substrate intermediates in the enzyme-catalyzed reaction pathway. Based on kinetic data and pH-rate profiles, the histidine (H115/H134) dyad is also proposed to play an important role in acid-base catalysis [29]. The histidines presumably activate a water molecule by its deprotonation through a proton transfer mechanism (Figure 2). However, the hydroxide ion undergoes a nucleophilic attack at the carbonyl functional group, which results in an unstable tetrahedral intermediate with a short lifetime. The attacking hydroxide ion seems to interact not only with H115 but also with glutamic acid E53 [30]. Comparisons of different substrate docking models [30] together with computational theoretical studies also suggest a potential role for aspartic acid D269 [31,32].

**Figure 3:**
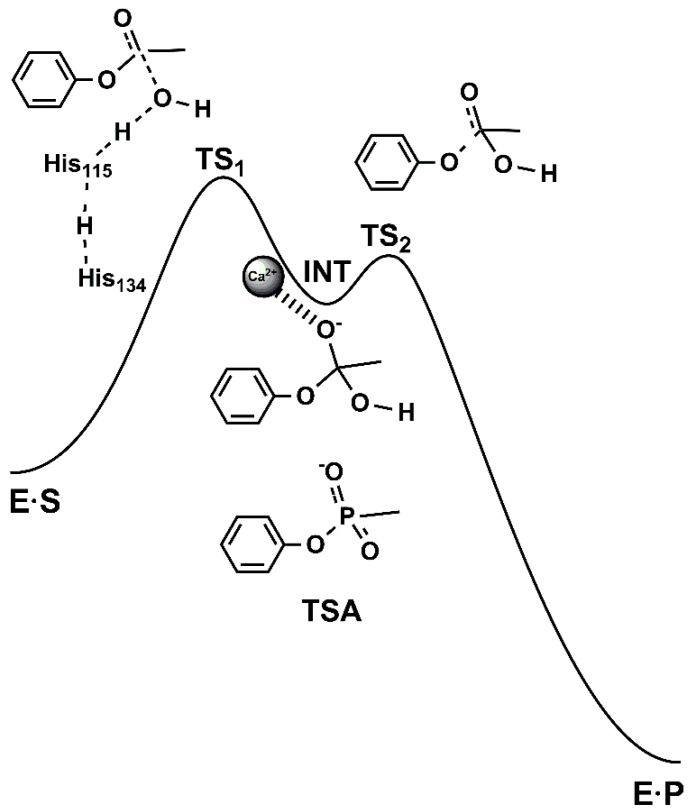
The generally accepted addition-elimination mechanism of PON1-catalyzed ester hydrolysis in the reaction pathway between the enzyme-substrate (ES) and enzyme-product (EP) complexes. The phosphonate-based ligand as a transition state analogue (TSA) should mimic the transition states (TS1 and TS2) near the tetrahedral intermediate (INT).

The negative charge of the resulting intermediate (and respective transition states) (Figure 3) can be stabilized by the catalytic calcium ion. However, a completely different pattern is observed for aryl versus aliphatic esters. Structure-reactivity studies show the absence of dependence on the leaving group pK_a_ (≈7.1–10.3) for aryl esters. Therefore, one can conclude that PON1 arylesterase rates are limited by a final physical step (i.e., product release or a conformational change in the protein) and not by chemical bond-making/-breaking processes. It should also be emphasized that the hydrolysis of PA (e.g., one of the best substrates of PON1) is diffusion-controlled, as demonstrated by viscosity experiments. In contrast, the hydrolysis of aliphatic acetate esters is obviously much slower than the hydrolysis of phenyl with a catalytic efficiency (k_cat_/K_M_) of <10^2^ M^−1^ s^−1^. However, acetate aliphatic esters show a sensitivity to the leaving group pK_a_ (≈12.5–16.1) that is similar to hydroxide-catalyzed nonenzymatic hydrolysis in aqueous solution [19].

Temperature dependence studies on the binding and catalysis of the arylesterase activity of PON1 have suggested the following: (i) the rate-limiting step is the nucleophilic attack of the water molecule on the carbonyl group of PA; (ii) the induced fit of the active site does not play an important role for arylesterase activity; (iii) the calcium ion bound to the active site is necessary for stabilizing the substrates and transition states [33]. Additionally, the effects of the solvent kinetic isotope on the PA esterase activity of PON1 indicate that two protons contribute to the rate-limiting process of the reaction route, while moderate inhibition with the phenyl methylphosphonate anion, which is a stable isosteric analogue (i.e., transition state analogue) (Figure 3) that mimics the high-energy tetrahedral intermediate in the hydroxide-promoted hydrolysis pathway, reveals that the transition state suboptimally resembles the tetrahedral adduct [34]. Thus, all the mechanistic details of the reaction pathway of PON1-catalyzed ester hydrolysis remain unclear.

**Figure 4:**
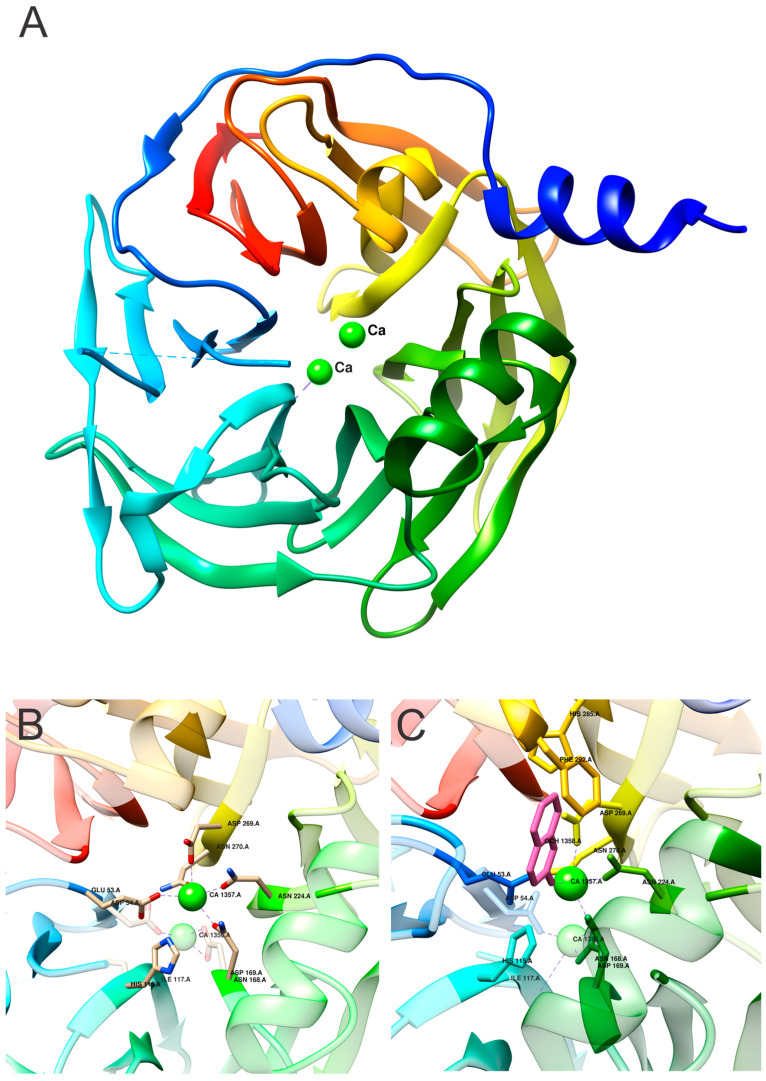
Paraoxonase 1 structure and insight into its active site. (**A**,**B**) Wild-type protein (protein database (PDB) id. 1V04 [28]). (**C**) Wild-type protein with bound inhibitor 2HQ (PDB id. 3SRG [30]). The active site residues are presented as backbone and marked by type and sequence number. Inhibitor is shown in magenta.

DHC is a lactone and another nonphysiological substrate for PON1 with high catalytic efficiency (k_cat_/K_M_ ≈ 10^6^ M^−1^ s^−1^) [19]. Its structural analogue is 2-hydroxyquinoline (2HQ), which is the best competitive inhibitor for all three enzymatic activities with similar micromolar inhibition constants. The crystal structure of the PON1 complex with 2HQ was obtained and revealed how 2HQ is bound to the catalytic calcium ion (Figure 4C) [30]. However, noncatalyzed enthalpy changes for DHC hydrolysis are 8 kJ/mol more exothermic than for PA hydrolysis, which indicates that the lactones are strained relative to the acyclic esters [35]. The implication of thermodynamic data regarding PA and DHC is that the latter exhibits greater steric strain in the ground state. The observations of enthalpy changes and rate data of PA and DHC in aqueous solutions (Table 1) are consistent. DHC is more highly strained than PA and, consequently, the transition state for DHC hydrolysis is more easily achieved than the transition state for PA hydrolysis in aqueous solutions without enzyme.

The heats of hydrolysis deal with enthalpy differences between reactants and products and are independent of (catalyzed or noncatalyzed) reaction pathways, while rate measurements are concerned with energy differences between reactants and transition states and are highly dependent on the reaction mechanisms. Although there is a relatively small (two-fold) difference between the catalytic efficiency of PON1 for PA and DHC, opposite differences can be observed between turnover numbers, enzymatically noncatalyzed rates and enzyme-substrate affinities. Consequently, it is not possible to conclude that the arylesterase and lactonase activities of PON1 are catalyzed through the same reaction pathways. These mechanistic details are still enigmatic but it is believed that serum PON1 is most likely a lactonase.

The true physiological substrates for PON1 are not known but presumably HCTL could be one of them. Rate measurements in aqueous solutions have shown that k_OH_ ≈ 14 M^−1^ s^−1^ for HCTL with a protonated amino group [38]. There are two putative models of anchimeric assistance of the amino group in HCTL: (i) intramolecular, general base catalysis of the deprotonated −NH_2_ functional group, and (ii) intramolecular, general acid catalysis of the protonated −NH_3_^+^ functional group. However, PON1 seems to be a very inefficient enzyme for thiolactonase activity in the case of HCTL, with a catalytic efficiency constant (k_cat_/K_M_) of only approximately 10 M^−1^ s^−1^ [1]. For comparison, the catalytic efficiency of PON1 is 500-fold higher (k_cat_/K_M_ ≈ 5000 M^−1^ s^−1^) in the case of the five-membered cyclic lactone analogue γ-butyrolactone. Both five-membered cyclic esters (γ-butyrolactone and HCTL) show very low but similar affinities for binding to the active site of PON1 (>20 mM) but, consequently, turnover numbers for enzyme-catalyzed hydrolysis of these two substrates indicate a high discrepancy. The observations of rate data would be in conflict if the reaction pathways were suggested to be similar for both substrates. In such a case, quite opposite values would be expected for the k_cat_/K_M_ ratio, as thioester bonds are energetically richer than ester bonds.

The name PON1 is historically derived from paraoxon, although this phosphate triester is hydrolyzed by PON1 with only modest catalytic efficiency (k_cat_/K_M_ ≈ 6000 M^−1^ s^−1^). Paraoxon represents P-O rather than C-O bond cleavage (and a pentavalent rather than a tetrahedral oxyanionic intermediate) during hydrolysis. Structure reactivity studies have shown that the electronic effect of the leaving group (β_LG_) plays different roles in PON1 and nonenzymatic (hydroxide-catalyzed) hydrolysis of phosphotriesters. Together with a rather low turnover number (k_cat_ ≈ 5 s^−1^), Khersonsky and Tawfik [19] concluded that these observations are inconsistent with the hypothesis that PON1 exhibits native phosphotriesterase activity.

## 4. Structural Insight into the Active Site of PON1

PONs are calcium-binding enzymes [39]. The two calcium ions have different functions; the more tightly bound ion is the so-called structural calcium ion (Ca2), and its removal causes irreversible structural disruption [28]. The other ion is the so-called catalytic calcium ion (Ca1), and its reversible removal causes a loss of catalytic activity that is recovered after the addition of calcium ions [39]. Ca1 interacts with the side-chain oxygens of N224, N270, N168, D269, and E53 and functions as an oxyanion that stabilizes the substrate and is necessary for optimal hydrolytic activity.

PON1 retains the signal peptide that is usually removed after protein translocation and is likely related to the protein property of binding to HDLs [40]. PON1 is also highly glycosylated, which makes it difficult to produce recombinantly. The first attempts to produce recombinant PON1 in *E. coli* resulted in aggregated proteins [40,41]. The soluble form of rePON1 produced in *E. coli* was obtained by DNA shuffling of human, mouse, rat and rabbit *PON1* genes. The resulting gene is used for structural studies and exhibits larger sequence identity to rabbit PON1 than human PON1 [1]. Several variants were designed and tested for the hydrolysis of PA and paraoxon. PON1 produced in bacteria retains its activity; therefore, glycosylation is not essential for its hydrolytic activity. PON1 has four potential glycosylation sites, and only two of them (N253 and N324) are located on the protein surface and are thus accessible for N-glycosylation by glycosidases [28].

The production of soluble rePON1 enabled structural studies and, therefore, detailed insight into the active site of PONs. Crystal structure revealed a six-bladed β-propeller with a unique active site lid (Figure 4A), which was shown to be involved in the HDL binding of the enzyme [28]. The structure was solved by using Se-Met protein and isomorphous replacement because of the lack of similar proteins to be used as a model for molecular replacement. The N-terminal part, i.e., the signal peptide, is missing in the structure, although it is known that the signal peptide is not cleaved. One surface loop is also missing. Beta-blade propellers with more than four blades are rare but conserved throughout the PON family, and each blade contains four strands. The characteristic closure of the fold is complemented by the disulfide bridge connecting N- and C-terminals of the protein (C42 and C353), and the replacement of these two cysteines leads to PON1 inactivation [42]. PON1 has one additional free cysteine residue (C284), and its substitution to alanine results in a modest decrease in paraoxonase and arylesterase activities [42]. Moreover, C284 is essential for the function of PON1 in protecting LDLs from oxidation [43]. Both calcium ions are in the central tunnel of the propeller on top of each other. The three paraoxonase, arylesterase and lactonase activities of PON1 share the same active site [28,29]. Histidines (H115, H134, H155, H243, and H285), tryptophan (W281), and aspartic acid/glutamic acid residues (D54, D169, D183, D269, D279, E53, and E195) are involved in the arylesterase and paraoxonase activities of PON1 [42]. The H115/H134 dyad mediates both lactonase and arylesterase activities. Mutations in one or both histidines reduce or completely abolish both activities [29]. Interestingly, the substitution of H115 to tryptophan results in an increase in paraoxonase activity toward paraoxon and the nerve agent VX [44]. In addition, phenylalanine at position 222 affects paraoxonase but not arylesterase activity [45]. Ca1 seems to interact with N224, N270, N168, N269, and E53, which are all located within a 2.5 Å radius. Higher solvent accessibility of Ca1 compared to that of Ca2 indicates that Ca2 is the higher-affinity calcium ion (Figure 4B). This correlates with the observations that removing this ion irreversibly destroys the protein structure [28].

In the crystal structure, there is only one molecule per asymmetric unit, suggesting that crystallization may favor the monomeric form. It was shown that the oligomeric state of human plasma isolated hPON1 is highly affected by the type and concentration of detergent in the buffer. However, hPON1 arylesterase activity is not detergent-dependent. Detergent dependence is typical for membrane proteins, and although PON1 is not a membrane protein, it exhibits similar properties, most likely due to its localization, i.e., association with HDLs [41]. The same behavior has been demonstrated for other HDL-bound proteins, including apoA-I and cholesteryl ester transfer protein. The oligomerization of PON1 is likely the consequence of its anchoring to detergent micelles in a mode similar to its anchoring to HDLs [28]. The N-terminal part of PON1 is responsible for HDL binding and behaves like a transmembrane helix. The residue 192, related to the two most common isoforms of the protein R192Q, is located in the active site area, which explains its strong influence on protein activity and function. Additionally, the mutation L55M may affect protein stability and, therefore, indirectly affect protein activity [28]. Only the loops that connect the β-strands define the function of most proteins with a β-propeller structure, defining the active site architecture and anchoring to HDLs [28].

To date, several structures of PON1 have become available. One of them is in complex with the competitive inhibitor 2HQ (Figure 4C). 2HQ lactam is a nonhydrolyzable lactone analogue in which esteric oxygen is replaced by NH. The corresponding lactone DHC is normally hydrolyzed by PON1 and is used as a substrate to test protein activity [30]. The binding of the inhibitor does not cause any significant changes to the structure of the active site. 2HQ does not interact only with Ca1 in the active site but also with the side chains of H115, D269, E53, and N168 (Figure 4C).

Crystal structures revealed that PON1 has at least three different active site conformations based on the position of the loop region: (i) closed conformation with a bound inhibitor (Figure 4C) in which the active site loop is structured and anchored to the enzyme’s surface, and Y71 points into the active site; (ii) opened—unbound conformation (structure determined at pH 4.5, Figure 4A,B) in which the active site loop is flexible (and thus not visible in the crystal structure), but Y71 is fixed and points outside of the active site; (iii) opened—unbound conformation (structure determined at pH 6.5, not shown) in which the loop including Y71 is flexible. All described conformations provide different interaction potentials and may mediate different catalytic activities [30].

**Figure 5:**
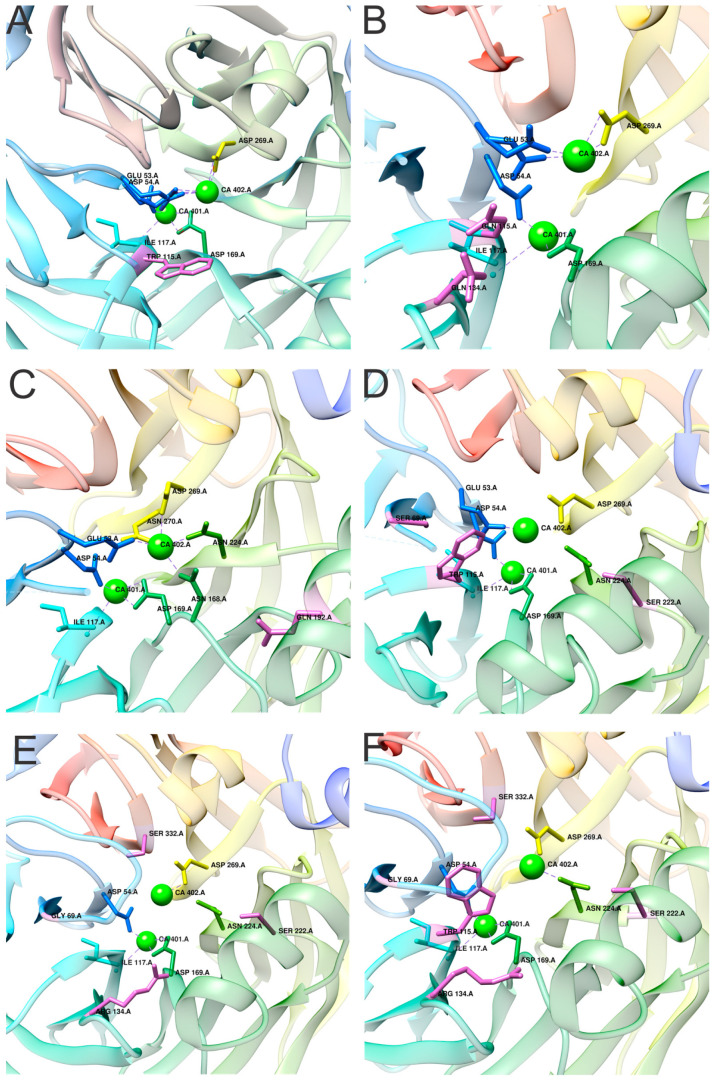
Paraoxonase 1 structural insight into active site of mutated proteins. Introduced mutations are related to protein specificity, activity or evolution. (**A**–**C**) Protein mutants related to enzymatic activity. (**A**) A mutant exhibiting only OP activity (H115W; PDB id. 4HHO [46]). (**B**) A mutant exhibiting convergence between OP and lactonase activity (H115Q and H134Q; PDB id. 4HHQ [46]). (**C**) A mutant affecting HDL binding and active site geometry (K192Q; PDB id. 4Q1U [47]). (**D**–**F**) Mutations related to the discovery of evolutionary trajectories. (**D**) L69S, H115W, and F222S (PDB id. 6G82 [49]); (**E**) L69S, H134R, F222S, and T332S (PDB id. 6GMU [49]); (**F**) L69S, H115W, H134R, F22S, and T332S (PDB id. 6H0A [49]). Mutated residues are in magenta.

Ca1 still retains limited flexibility and can rearrange inside the active site. It was shown that the alternative modes of Ca1 are related to lipo-lactonase catalytic promiscuity and divergence into PON [46]. The mutation of H115 (an active site residue) (Figure 5A) to tryptophan diminishes lactonase activity and concurrently enhances paraoxonase activity. Crystal structure revealed that the mutation of H115 into tryptophan displaces Ca1 for 1.8 Å towards the enzyme surface. The same Ca1 translocation occurs when both H115 and H134 (Figure 5B) are mutated to glutamine. These mutations abolish 2HQ binding to the enzyme, which correlates with the loss of lactonase activity [46].

The stability and catalytic activities of PON1 are highly stimulated when the enzyme associates with HDLs. This is likely due to the network of hydrogen bonds between the binding site (that binds to HDLs) and the active site, even though there is >15 Å between them (Figure 5C). This network of hydrogen bonds aligns the lactone substrate with Ca1 [47]. Three residues have been identified that enable complex formation between HDLs and PON1 L9, Y185 and Y293 [48].

PON1 was used to study the evolutionary trajectories of enzyme functions. Due to evolutionary changes, ancient functions are usually lost. By studying active site mutations, the trajectories between lactonase and paraoxonase activity were studied; each exhibited tendencies towards different substrates and reactions but started with the same residue, H115, in the active site. Residue loss resulted in lactonase activity loss and, moreover, increased paraoxonase activity [46]. The neofunctionalization trajectory amplifies paraoxonase activity, and the re-functionalization trajectory restores and therefore amplifies lactonase activity. The latter generates new lactonases that lack H115 active site residues. The mutations L69S, H115W, and F222S enhance paraoxonase activity (the lactonase activity decreased by >5000-fold) (Figure 5D). The most active PON variant is the one bearing five mutations in the active site (L69S, H115W, H134R, F222S, and T332S) (Figure 5F). Using the enzyme with five mutations (the most active enzyme) and reverting W115 back to the original histidine resulted in the loss of both lactonase and paraoxonase activity (Figure 5E) [49].

## 5. Paraoxonase 2: The Oldest Member of the Family

PON2 is a ubiquitously expressed intracellular enzyme, which has been detected in several tissues in humans and animals [50,51,52]. The expression of PON2 exhibits gender differences, as female mice exhibit higher PON2 expression in nerve tissue and higher lactonase activity [50]. The product of the *PON2* gene is a protein composed of 354 amino acid residues with a molecular mass of 40–43 kDa. PON2 has three potential N-glycosylation sites (N252, N269, and N323) [53], and N252 and N323 are present at homologous positions in PON1 (N253, N324) [28]. The additional N-glycosylation site might explain the different molecular masses of PON2 [26]. Modelling PON2 structure reveals very high structural similarity to PON1 [54]. Studies have shown that PON2 is localized to the membrane of the endoplasmic reticulum and mitochondria and to the plasma membrane [55,56,57,58]. Mitochondria are the main source of free radicals, and thus the localization of PON2 to this organelle suggests its protective role against oxidative stress. PON2 binds to coenzyme Q10 and prevents mitochondrial superoxide formation independently from its lactonase activity [55,56]. In several tissues, PON2 exhibits antioxidant properties by reducing cellular oxidative damage, promoting redox potential, and preventing apoptosis [55]. It should be noted that among the three PONs, PON2 is the only one expressed in nervous tissues, and high PON2 levels in the brain protect neurons against lipid peroxidation and oxidative stress toxicity [50]. These antioxidant effects also play a significant role in preventing the atherosclerotic process [59] and heart failure [60]. Compared to PON1 and PON3, PON2 shows no paraoxonase activity, very low arylesterase activity, low lactonase activity towards DHC and the highest lactonase activity towards N-acyl-homoserine lactones [26,61]. In this respect, PON2 is the most potent quorum quencher, which efficiently degrades bacterial biofilms and slows down pathogenesis of peritonitis in a mouse model [62]. Its catalytic activity depends on post-translational modifications on the N-terminus, such as glycosylation [55,63]. The ubiquitination of lysine 168 is responsible for the decrease in PON2 arylesterase activity [64]. One of the common isoforms of PON2, with a Ser/Cys substitution at position 311, results in decreased lactonase activity [63]. Lower lactonase activity is associated with a higher risk for myocardial infarction, diabetes mellitus and Alzheimer’s disease [65,66,67]. Furthermore, PON2 deficiency leads to an increased susceptibility to obesity [68]. PON2 not only has an enzymatic function but also acts in cellular signaling as a regulator of epithelial Na^+^ channels [69], an activator of the PI3K/Akt/GSK-3β RISK pathway [70], a facilitator in GLUT1-mediated glucose transport [71], a tumor suppressor in ovarian cancer [72] and a regulator of coagulation activation [73].

## 6. Paraoxonase 3: In the Shadow of his Younger Brother

**Figure 6:**
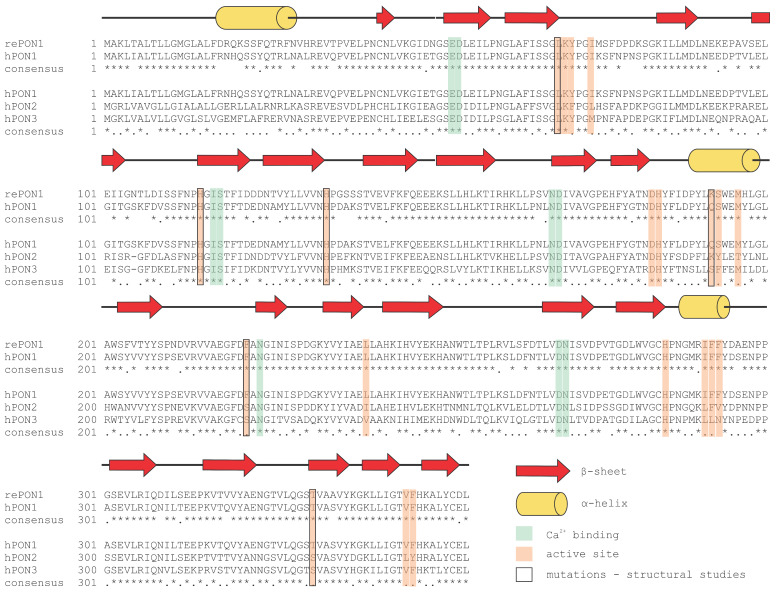
Sequence alignment of recombinant PON1 (rePON1) and human PON1 (hPON1). The rePON1 protein sequence used for structural studies is from the protein database—PDB id: 1V04. The hPON1, hPON2, and hPON3 sequences are from the UniProt database—P27169, Q15165, and Q15166, respectively. In consensus line (*) is annotating conserved sequence, (**.**) is annotating conservative mutations and ( ) is annotating nonconservative mutations. Multiple sequence alignment was performed by T-Coffee, and Boxshade was used for alignment presentation. Secondary structure annotation, Ca^2+^ binding sites (both structural and catalytic ion), and active site residues are adapted from (as marked by different colors) [28]. The point mutations that were also evaluated by structural studies are annotated according to PDB id sequences: 4HHO, 4HHQ [46], 4Q1U [47], 6G82, 6GMU, and 6H0A [49].

PON3 is synthesized in the liver and, to a lesser extent, in the kidney. It is mainly associated with HDLs in the blood [4] but is also present in mitochondria [74,75]. PON3 is a 40–45 kDa glycoprotein with 354 amino acid residues that requires a calcium ion for its catalytic activity [4,5]. Compared to PON1, PON3 exhibits low arylesterase activity, high lactonase activity and no paraoxonase activity (except paraoxon hydrolysis). Interestingly, PON3 exclusively hydrolyzes bulky statin lactones, including lovastatin, spironolactone, canrenone and atorvastatin [26,76], which are commonly used to monitor PON3 activity due to their specificity towards PON3. However, the structure of PON3 has not been determined yet. As PON3 poorly hydrolyzes simple lactones or lactones with small, polar substituent groups, yet exhibits similarities with PON1 regarding its active site amino acid residues, it has been hypothesized that the active site of PON3 is larger than that of PON1 [77,78]. However, the structural geometry of the active site might differ from the predicted one. Two amino acid residues in the active sites of PON1 and PON2, tryptophan at position 281 (W281) and histidine at position 243 (H243), are substituted by leucine and lysine, respectively, in human PON3 (Figure 6) [79]. Mutational studies showed that W281 and H243 are involved in the ability of PON1 to hydrolyze aryl esters; their absence could explain the very low arylesterase activity of PON3 [42].

PON3 is approximately 200-fold less abundant than PON1 in rabbit serum but 100-fold more efficient in protecting LDLs against lipid oxidation [4]. The development of many diseases is associated with changes in PON3 levels. Increased serum levels of PON3 are linked with its protective role in chronic liver impairment [80], coronary artery disease [81], and chronic kidney disease [82]. In male mice, it has been demonstrated that an increase in liver PON3 activity significantly decreases atherosclerotic lesion formation and adiposity [83]. Thus, we may speculate that increased PON3 concentration and activity in the above-mentioned diseases may be enough to compensate decreased PON1 levels and their resulting negative, pathological effects. Additionally, PON3 interacts with coenzyme Q10 and protects against mitochondrial oxidative stress-mediated cell death [74]. However, knockout mice models have demonstrated that PON3 expression remains unchanged in response to oxidative stress [84].

## 7. Paraoxonase Versatility

The human *PON-1*, *-2*, and *-3* genes are located very close to each other on the long arm of chromosome 7 (7q21.3–q22.1), and each of them consists of nine exons and eight introns. A comparison of PON nucleotide and amino acid sequences demonstrated approximately 70% and 60% identity, respectively [85]. *PON1* differs from *PON2* and *PON3* in that it has three additional nucleotide residues in exon 1, and lysine is additionally present at codon position 106 in human PON1 [86]. Although all three enzymes exhibit very similar amino acid sequences (Figure 6), they exert different functions depending on their location in the human body (Table 2). Nevertheless, all PONs are characterized by their protection against oxidative stress. The evolutionarily oldest member of the family appears to be PON2, from which PON3 and the youngest PON1 have evolved, presumably as a result of gene duplication during evolution [25]. Although the name derives from the ability of PON1 to hydrolyze paraoxon [2,3], PON2 and PON3 do not exhibit obvious paraoxonase activity and were named the same only because of their evolutionary relationship to PON1 [87]. All three PONs exhibit arylesterase activity but apart from PON1, these activities are very low. They also act as lactonases with some overlapping substrates (like aromatic lactones) yet also exhibit distinct substrate specificities [26]. The native substrate of all three PONs is N-acyl homoserine lactone, which is involved in quorum sensing, the communication strategy between microorganisms. The physiological substrates of PONs are poorly understood, yet recent findings shed light onto their structural requirements. Two bioactive δ-lactones derived from arachidonic acid, 5,6-dihydroxy-eicosatrienoic acid lactone and cyclo-epoxycyclopentenone, were efficiently hydrolyzed by PON1 and PON3 [88]. PON1 and PON3 reside in HDLs, whereas PON2 is an intracellular enzyme. All three PONs are calcium-dependent hydrolases.

Human PONs have hydrophobic N-terminal regions. However, these regions contribute to binding with blood plasma HDLs only in PON1 and PON3 [28]. PON1 levels in the blood and genotype-related catalytic activity have a major impact on an individual’s susceptibility to OPs (pollutants and insecticides), development of atherosclerosis and drug metabolism [16,18,25].

Structural data are available only for PON1 and its mutants. We termed the variant used for structural studies as rePON1, and this sequence was also used for sequence alignment. The annotated secondary structure is based on the rePON1 sequence. Several mutations to the sequence have been applied in order to change the enzyme specificity or to explain the evolutionary trajectories [1,24,28,30,46,47,49].

Medical applications involving human paraoxonases are under development. PON1 has potential as an antidote in acute OP poisoning. The engineering of soluble enzyme with improved OP activity and produced in bacteria is of high interest [10,11,23,24,93]. Additionally, since PON1 is produced in the liver only and, therefore, its expression level and detected activity decreases with liver damage, it has the potential to be used as a prognostic marker in hepatocellular carcinoma [94,95], liver disorders [96] and liver transplantation patients [97]. PON2 efficiently degrades N-acyl homoserine lactones and has, therefore, potential as an enzymatic alternative to conventional antibiotic treatment. The increase of antibiotic-resistant pathogenic bacteria requires urgent development of alternative strategies to fight against them. The development goes also in the direction of using enzymes that can affect quorum-sensing systems [98]. PON3 is mainly expressed in the liver, has a cell proliferation inhibitory function and may serve as a prognostic predictor or therapeutic target in patients with hepatocellular carcinoma and esophageal cancer [99,100].

The positive role of PON1 in OP poisoning, its protective role in cardiovascular diseases and the large difference in PON1 expression and activity between the individuals, is opening the question of finding a good agonist that would minimize difference, or be used as supportive or acute drug, in situations where increased activity is of high importance [101]. In the case of PONs, the identification of inhibitors is not the drug development process against PON but the opposite, i.e., it is important to identify inhibitors (potential or already used drugs) to regulate their side effects that result from PON inhibition [102,103,104,105,106,107].

## 8. Conclusions and Future Perspectives

Different PON1 activities that all reside in the same active site enable evolutionary studies to determine the point of divergence. Furthermore, the known PON1 structure and high sequence similarity to PON2 and PON3 provide starting points to study PON2 and PON3 activity and their specificity for substrate type and size. Nevertheless, data on the structure and function of PONs is still missing. Thus, future work should focus on several pertinent issues of which some of the most obvious are listed here.

1. Structural data on PON2 and PON3 could explain the substrate preferences of all three PONs.
2. Several studies have reported either higher or lower activity of predominantly PON1 in different diseases. However, it is still unclear whether elevated PON1 levels cause disease or represent a protective mechanism related to their antioxidative properties.
3. The primary substrate of all three enzymes remains to be confirmed.
4. Additional studies on point mutations could improve paraoxonase activity, which could enable the use of PON1 as an antidote in acute OP poisoning.
5. Searching for PON agonists is of high importance in prevention strategies of development of cardiovascular diseases.
6. Studies focused on testing already approved drugs and their effect on PON activity may explain the side effect and further improve the treatment of various conditions.
7. Although PONs are relatively small proteins, their binding to membranes and HDLs could be studied using artificial membranes. As studies report that binding to membranes (also in the presence of detergents) can promote PON oligomerization, cryoelectron microscopy could help elucidate this phenomenon.

## Figures and Tables

**Table 1:** Enthalpy (ΔH) and rate (k_OH_) changes for the alkaline hydrolysis of phenyl acetate, dihydrocoumarin, and paraoxon. k_OH_ is the second-order rate constant for the hydroxide ion reaction with the carbonyl groups of various substrates. k_cat_, K_M_ and k_cat_/K_M_ are the turnover number, the Michaelis constant and catalytic efficiency, respectively. ^a^ [35]; ^b^ [19]; ^c^ [36,37].

Substrate	ΔH (kJ/mol)	k_OH_ (M^−1^ s^−1^)	k_cat_ (s^−1^)	K_M_ (mM)	k_cat_/K_M_ (M^−1^ s^−1^)
phenyl acetate	−97 ^a^	1.3 ^a^	700 ^b^	1.2 ^b^	580,000
dihydrocoumarin	−105 ^a^	824 ^a^	150 ^b^	0.13 ^b^	1,150,000
paraoxon	n.d.	0.075 ^c^	4.8 ^b^	0.8 ^b^	6000

**Table 2:** A comparison of all three paraoxonases.

	PON1	PON2	PON3
UniProt id	P27169 (PON1_HUMAN)	Q15165 (PON2_HUMAN)	Q15166 (PON3_HUMAN)
gene locus	long arm of chromosome 7 (7q21.3-q22.1) [85]		
	9 exons and 8 introns [85]		
number of aminoacids	355 insertion of K106 [86]	354	354
theoretical molecular weight	39.7 kDa	39.4 kDa	39.6 kDa
size from SDS-PAGE	43 kDa [89]	40–43 kDa [90]	40–45 kDa [4,5]
calcium-dependent glycoprotein	yes [89]	yes	yes [4,5]
hydrophobic N-terminal region	yes [28]	yes [57]	yes
confirmed N-glycosylation sites	N253, N324 [91]	N254, N323 [55,63,64]	N323 [92]
other post-translational modifications	n.d.	ubiquitination of K168 [64]	n.d.
3D structure (PDB id)	1V04 [28], 4HHO [46], 4HHQ [46], 4Q1U [47], 6G82 [49], 6GMU [49], 6H0A [49], 3SRG [30], 3SRE [30]	n.d.	n.d.
EC classification	3.1.1.2 3.1.1.81 2.1.8.1	3.1.1.2 3.1.1.81	3.1.1.2 3.1.1.81 2.1.8.1
protection against oxidative stress	yes [10]	yes [55,59]	yes [74,84]
arylesterase activity	yes [18]	yes very low [26,61]	yes low [26,76]
lactonase activity	yes [19]	yes low against DHC [26,61]	yes high [26,76]
organophosphate activity	yes [23,24]	no [26,61]	yes paraoxon only [26,76]
synthesis	produced in the liver [8]	ubiquitously expressed [50,51,52]	produced in the liver and to a lesser extent in the kidney [4]
location	mainly bound to HDLs in blood plasma in complex with myeloperoxidase [7]	intracellular enzyme on the membrane of mitochondria on the ER on the plasma membrane [55,56,57,58]	mainly bound to HDLs in blood plasma and mitochondria [4,74,75]

## Abbreviations

Ca1	catalytic calcium
Ca2	structural calcium
DHC	dihydrocoumarin
HCTL	homocysteine thiolactone
HDLs	high-density lipoproteins
hPON1	human PON1
hPON2	human PON2
hPON3	human PON3
kDa	kilodaltons
k_cat_	turnover number
K_M_	Michaelis constant
k_OH_	second-order rate constant for the hydroxide ion reaction with carbonyl groups of various substrates
LDLs	low-density lipoproteins
OP	organophosphate
PA	phenyl acetate
PDB	protein database
PON1	paraoxonase 1
*PON1*	paraoxonase 1 gene
PON2	paraoxonase 2
*PON2*	paraoxonase 2 gene
PON3	paraoxonase 3
*PON3*	paraoxonase 3 gene
rePON1	recombinant PON1
